# Use of Human Serum Albumin in Critically Ill Patients: A Narrative Review

**DOI:** 10.3390/jcm15051981

**Published:** 2026-03-05

**Authors:** Iñigo Rubio-Baines, Luigi Camporota, Duilio González-Delgado, Gemma Echarri, Maria Carmen Sala-Trull, Pablo Montero-López, Marc Vives

**Affiliations:** 1 Department of Anesthesiology and Critical Care, Clínica Universidad de Navarra, 31008 Pamplona, Spain; irubiob@unav.es (I.R.-B.);; 2 Department of Adult Critical Care, Guy’s and St Thomas’ NHS Foundation Trust, King’s Health Partners, London SE1 9RT, UK; 3 Division of Asthma, Allergy and Lung Biology, King’s College London, London SE1 7EH, UK; 4 Instituto de Investigación Sanitaria de Navarra (IdiSNA), 31008 Pamplona, Spain

**Keywords:** human serum albumin, critical care, hypoalbuminemia, fluid therapy

## Abstract

**Background**: Human serum albumin (HSA), the most abundant plasma protein, is essential for oncotic pressure, endothelial protection, drug binding, and immune modulation. Despite its widespread clinical use since the 1940s, its therapeutic benefit in critically ill patients remains debated. This narrative review summarizes current evidence on HSA use in common intensive care scenarios. **Clinical Applications**: In hepatorenal syndrome (HRS), albumin combined with vasoconstrictors like terlipressin improves renal function and survival. In spontaneous bacterial peritonitis (SBP), albumin lowers the risk of acute kidney injury and mortality, particularly in high-risk cirrhotic patients. Post-paracentesis albumin reduces circulatory dysfunction and may enhance survival in cirrhosis. For septic shock, trials show no overall mortality benefit over crystalloids, though albumin may offer hemodynamic advantages in specific subgroups. In acute respiratory distress syndrome (ARDS), albumin improves oxygenation in hypoalbuminemic patients, without survival benefits. During major cardiac or abdominal surgery, albumin reduces fluid needs and postoperative complications, especially in hypoalbuminemic individuals. In acute brain injury, albumin’s role is controversial: it may aid recovery after cerebral hemorrhage, but can worsen outcomes in traumatic brain injury. In trauma and ECMO patients, albumin may stabilize hemodynamics and improve outcomes in selected cases. **Conclusions**: Inappropriate albumin use remains common, and evidence on its optimal concentration, dose, timing, and patient selection is limited. HSA is safe and beneficial in specific situations. Routine use should follow evidence-based guidelines. Future research must identify patients who are most likely to benefit and clarify optimal dosing strategies, concentrations, and therapeutic goals.

## 1. Background

Albumin is a 67 kDa protein composed of 585 amino acids. It is synthesized by hepatocytes but not stored within them, and the body has no organic reserve of albumin [1]. Although the total mass of albumin is greater in the extravascular compartment, its concentration is higher in serum [2]. Among its functions, human serum albumin (HSA) maintains oncotic pressure, supports the endothelium and glycocalyx integrity, and has anti-inflammatory properties. It also influences pharmacokinetics and pharmacodynamics by binding a wide range of drugs and substances, and plays an important role in maintaining physiological homeostasis [2,3,4,5]. Several preparations of HSA exist, the most common being 20% and 5% solutions, the safety of which has been evaluated [6,7,8]. Although albumin synthesis may increase during critical illness [9], critically ill patients frequently develop hypoalbuminemia, defined as a serum albumin concentration below 30 g/L. Potential causes include fasting, gastrointestinal losses, hemorrhage, or hemodilution secondary to fluid resuscitation. Hypoalbuminemia is associated with worse outcomes in critically ill patients, although it is uncertain whether this is a direct effect or simply a reflection of the severity of critical illness [7]. When the integrity of the endothelial glycocalyx is maintained, albumin solutions remain intravascular longer than crystalloid solutions, largely due to their molecular weight [10,11]. However, when glycocalyx integrity is compromised, albumin may move into the extravascular space faster, leading to tissue edema. HSA is predominantly used in hospital settings and, although various international organizations have issued guidelines for its use in different clinical situations, variations in practice and inappropriate administration remain common, especially in cases where there is insufficient clinical evidence supporting its use. The rate of incorrect albumin use varies across studies in different countries, ranging from 50% to 70%, and was found to exceed 90% in some studies [12,13,14]. The most common scenarios where HSA is used include: (a) End-stage Liver Disease (Hepatorenal Syndrome and Spontaneous bacterial peritonitis), (b) large-volume and modest-volume paracentesis, (c) septic shock, (d) acute respiratory distress syndrome (ARDS), (e) perioperative major surgery (cardiac and abdominal), (f) Acute brain injury, (g) trauma patients, (h) patients with extracorporeal membrane oxygenation (ECMO).

In this narrative review, we discuss the existing evidence regarding the use of albumin in critically ill patients in these scenarios.

## 2. Methodology

### 2.1. Literature Search Strategy

Data were gathered through a search of electronic databases to identify studies related to the clinical indications of albumin use published from 2010 through 2025. The search was conducted in PubMed, Google Scholar, Scopus, and Web of Science. The search terms and keywords included sepsis, septic shock, acute respiratory distress syndrome, mortality, hypoalbuminemia, plasma exchange, paracentesis, plasmapheresis, cardiac surgery, major surgery, trauma, cirrhosis, human albumin treatment or therapy, human albumin infusion, fluid resuscitation, and critical care. All studies included were human studies and published in English. As this was a narrative review, no formal systematic screening process (such as PRISMA) was applied, and study selection was based on relevance to the predefined clinical scenarios.

#### 2.1.1. End-Stage Liver Disease

In chronic liver disease, hypoalbuminemia is not merely a reduction in circulating albumin concentration but also reflects profound qualitative alterations in the protein itself, including oxidative damage and impaired binding capacity, which have been linked to liver dysfunction and worse survival in cirrhotic patients [15,16,17]. These structural modifications result in loss of albumin’s normal antioxidant, immunomodulatory, and transport functions, contributing to the hallmark pathophysiology of cirrhosis. Dysfunctional albumin and persistent hypoalbuminemia aggravate endothelial instability and increased vascular permeability, promote systemic inflammation by failing to buffer cytokines and reactive species, and disrupt the delicate coagulation–fibrinolysis balance by altering nitric oxide bioavailability and platelet interactions, all of which exacerbate circulatory and hemostatic derangements in advanced liver disease [17,18]. Moreover, clinical evidence consistently shows that albumin levels and functional integrity correlate with disease severity, circulatory dysfunction, and outcomes in cirrhosis, and that therapeutic albumin administration exerts beneficial effects beyond oncotic support by modulating vascular permeability, inflammatory signaling, and components of coagulation homeostasis [19,20].

- Hepatorenal syndrome

Hepatorenal syndrome (HRS) is a form of kidney dysfunction that develops in patients with cirrhosis and ascites. Although traditionally regarded as a functional disorder, it is now recognized to coexist with structural kidney injury, proteinuria, or pre-existing CKD. HRS results from a reduction in effective arterial volume due to splanchnic vasodilation, portal hypertension, renal vasoconstriction, and persistent systemic inflammation despite adequate fluid resuscitation. HRS may be classified as type 1 when acute renal dysfunction is precipitated by an acute event, or as type 2 when the renal decline is more gradual and associated with refractory ascites [21].

The 2023 ADQI criteria define HRS-AKI as an acute increase in serum creatinine and/or oliguria and absence of response to fluid therapy after 24 h, in patients with cirrhosis and ascites with no other cause of AKI. The routine use of albumin for 48 h is no longer needed for the diagnosis of HRS, allowing for the use of early vasopressors while avoiding the risk of fluid congestion [22]. In patients with decompensated cirrhosis, it is common to have concomitant AKI, which may progress to HRS and is associated with high mortality [23].

Data suggest a benefit from using a combination of albumin and vasopressors. An RCT on 196 patients with cirrhosis and HRS-1 showed that the use of terlipressin plus albumin was associated with better renal function, compared to albumin alone [24].

Another RCT of 300 patients with cirrhosis and type 1 HRS showed that albumin plus terlipressin was associated with a higher rate of HRS reversal than albumin alone (32% vs. 17%; *p* = 0.006). Furthermore, in a subanalysis of patients with cirrhosis and systemic inflammatory response syndrome (SIRS), the difference between groups was even higher (37% vs. 6%; *p* < 0.001) [25].

Furthermore, a meta-analysis of 13 RCTs involving 739 patients with HRS type 1 showed that albumin plus terlipressin was associated with a non-significant reduction in 3-month mortality compared with albumin alone (Table 1) [26].

In the meta-analysis by Nanda et al. [27], two main comparisons were performed. The first, including several randomized trials [24,28,29,30], showed that terlipressin plus albumin was superior to albumin alone in achieving HRS reversal. The second comparison, based on the study by Cavallin et al. [31], also demonstrated greater efficacy of terlipressin plus albumin compared with midodrine plus octreotide plus albumin. However, no significant benefit was observed with respect to HRS recurrence or mortality (Table 1).

Similarly, a network meta-analysis of 25 RCTs involving 1263 patients found that although albumin plus terlipressin did not significantly reduce mortality, it was associated with higher HRS recovery rates compared with albumin plus midodrine and octreotide or albumin plus octreotide alone (Table 1) [32].

In addition, a recent randomized trial in hospitalized patients with cirrhosis showed that additional albumin administration was associated with a trend toward lower rates of AKI and mortality compared with standard care (Table 1) [33].

Taken together, current evidence supports the use of albumin in combination with terlipressin in patients with HRS secondary to decompensated cirrhosis.

- Spontaneous bacterial peritonitis (SBP)

SBP is an ascitic fluid infection without an identifiable intra-abdominal source. It occurs most commonly in patients with cirrhosis and ascites. However, it may also occur in other conditions associated with ascites, such as heart failure, nephrotic syndrome, or malignancy [34,35,36]. Typically, SBP presents with fever, abdominal pain, altered mental status, and ascitic polymorphonuclear cell counts greater than 250 cells/mm^3^.

Based on the American Association for the Study of Liver Diseases guidelines, it is recommended, with a grade 1 A recommendation, to administer albumin for prevention of HRS in patients with high risk of HRS, including the following: (a) patients with total bilirubin level greater than 4 mg/dL, (b) patients with blood urea nitrogen (BUN) higher than 30 mg/dL, or serum creatinine above 1 mg/dL. The recommendation is to administer 25% albumin at 1.5 g/kg (up to 100 g/day) within 6 h of SBP diagnosis, followed by 1 g/kg (up to 100 g/day) on the third day [36].

Patients with SBP are at a higher risk of developing AKI (30–40%) due to activation of the renin–angiotensin system (RAAS) and a decrease in stroke volume (SV). The use of albumin has been associated with a lower rate of AKI and mortality in patients with SBP [37,38].

Therefore, based on the current evidence, it is recommended to use albumin for the prevention of HRS in patients at high risk of HRS.

#### 2.1.2. Large-Volume and Modest-Volume Paracentesis

Critically ill patients with severe cirrhosis and massive ascites are recommended to receive albumin infusion following large-volume paracentesis (LVP). The recommended dose is 8 g of albumin per liter drained (from the fifth liter). The rationale for albumin replacement is to reduce paracentesis-induced circulatory dysfunction (PICD).

LVP is currently the preferred treatment for cirrhosis-related massive ascites, and the addition of albumin infusion after LVP has been associated with lower rates of PICD and mortality. The ANSWER trial, which included 440 patients with cirrhosis and ascites, showed that albumin infusion reduced the risk of refractory ascites by 57% and mortality by 38%, compared with standard medical treatment alone. Furthermore, albumin infusion helped to prevent the need for subsequent paracentesis (HR: 0.48, 95% CI: 0.35–0.64) [39]. Additionally, data from a meta-analysis of 17 RCTs on 1225 patients with cirrhosis showed that albumin infusion after LVP was associated with lower rates of PICD (61% reduction) and mortality (36% reduction) [40]. However, a 2017 meta-analysis that included cirrhosis patients without hepatocellular carcinoma found no significant improvement in survival with albumin infusion following LVP (OR: 0.78, 95% CI: 0.55–1.11, *p* = 0.17) [41]. Similar results were observed in another meta-analysis by Simonetti et al. (*n* = 977, 27 RCTs, 2019) [42]. The differences in findings across these meta-analyses are most likely attributable to the inclusion of two controversial RCTs that compared albumin with diuretics and mannitol, respectively [43,44]. In the author’s opinion, excluding these two studies from the meta-analyses would most likely show a significant reduction in mortality with albumin use.

Based on current evidence, albumin infusion after LVP in critically ill cirrhosis patients with massive ascites is recommended to reduce the risk of refractory ascites, lower the incidence of PICD, and decrease the mortality rate.

#### 2.1.3. Severe Sepsis and Septic Shock

Multiple physiological mechanisms may elucidate why HSA administration may be beneficial in severe sepsis and septic shock. In addition to the expansion of intravascular volume, albumin has anti-inflammatory properties, promotes capillary and endothelial integrity, protects the glycocalyx, maintains acid–base homeostasis, and acts as a key binding protein for many antibiotics [45]. These properties have encouraged investigation into whether albumin brings benefits in relation to crystalloids in patients undergoing septic shock. There are two major RCTs assessing albumin in severe sepsis and septic shock: the SAFE and ALBIOS trials. The SAFE trial (Saline versus Albumin Fluid Evaluation) in 2004 randomly allocated nearly 7000 critically ill adults to receive 4% albumin or saline for fluid resuscitation. There was no significant difference in 28-day mortality with albumin in either group [6]. In the predefined severe sepsis subgroup (*n* = 1218), the trend of lower mortality after use of albumin was non-significant (RR 0.87, 95% CI 0.74–1.02). In adjusted multivariable analyses, albumin was associated with reduced mortality (adjusted OR 0.71, 95% CI 0.52–0.97; *p* = 0.03) [46]. The SAFE study showed that HSA is just as safe as saline for resuscitation, and suggested that it may improve survival in patients with severe sepsis. Still, the population included was primarily low-risk patients. The 2018 ALBIOS trial (Albumin Italian Outcome Sepsis) included 1818 patients with severe sepsis and compared 20% albumin infusion with crystalloid solution versus crystalloid solution alone. The target serum albumin concentration in the albumin group was ≥30 g/L until ICU discharge or 28 days post-randomization. Data showed no difference between groups in the primary outcome, 28-day mortality, with the albumin group data representing 31.8% and 32% (RR = 1, 95% CI 0.87–1.14, *p* = 0.94). The 90-day mortality within the two groups was similar (41.1% vs. 43.6%, RR 0.94, 95% CI 0.85–1.05, *p* = 0.29). Patients receiving albumin showed a higher mean arterial pressure (*p* = 0.03) and lower net fluid balance (*p* < 0.001). Moreover, a subgroup comparison of 1121 patients with septic shock revealed reduced mortality in patients treated with HSA solution (RR = 0.87, 95% CI: 0.77–0.99) [47].

Beyond its role as a plasma expander, HSA exerts several molecular and physiological effects that may help to explain the observed mortality benefit in the septic shock subgroup of the ALBIOS trial. HSA contributes to endothelial stabilization and preservation of the glycocalyx, thereby reducing capillary leak, interstitial edema, and microvascular dysfunction—key pathophysiological features of septic shock [2,11]. In addition, albumin has well-described antioxidant properties through binding and scavenging of reactive oxygen and nitrogen species, which may attenuate oxidative stress-induced cellular injury during systemic inflammation [2,7].

Albumin also modulates immune and inflammatory responses by binding endotoxins, cytokines, and damage-associated molecular patterns, potentially dampening excessive immune activation [2,7]. From a hemodynamic perspective, its colloid oncotic properties allow more effective intravascular volume expansion with lower net fluid balance, which may reduce venous congestion and organ dysfunction [46,47]. Furthermore, HSA plays a critical role in drug transport and pharmacokinetics, particularly for highly protein-bound antibiotics commonly used in sepsis, potentially optimizing antimicrobial exposure in hypoalbuminemic patients. Collectively, these pleiotropic effects may provide a biological rationale for the improved outcomes observed in selected patients with septic shock.

Other international recommendations are in accordance with the Surviving Sepsis Guidelines [48], limiting the use of albumin to patients requiring large volumes of crystalloids or when crystalloids alone are insufficient [45,48,49,50,51,52,53]. Expert opinion suggests that if hemodynamic instability continues after 30 mL/kg crystalloid resuscitation in patients with septic shock, the initiation of HSA infusion should be considered [48,49,50,51]. It is reasonable to consider initiating HSA infusion if hemodynamic instability persists despite 30 mL/kg of crystalloid and the patient remains fluid-responsive.

Thus far, no RCT has directly compared the efficacy and safety of varying concentrations of HSA solutions for fluid resuscitation in sepsis patients. Yet, large RCTs have included low-concentration (4% or 5%) and high-concentration (20% or 25%) HSA for resuscitation, with no serious adverse effects observed, suggesting that both concentrations can be safe in sepsis [6,46,47,52,53,54,55,56]. Various HSA concentrations were used across the different studies (4% albumin in the SAFE and RASP trials and 20% albumin in the ALBIOS trial) [6,47,53]. However, these larger trials did not compare outcomes with different levels of HSA. In a recent meta-analysis of 26,351 patients enrolled in 58 clinical trials, no significant difference was noted in mortality and total resuscitation fluid volume between low- versus high-concentration HSA solutions for patients with sepsis [57]. Taking these results into consideration, experts suggest that both low- and high-concentration HSA solutions for fluid resuscitation in sepsis patients are safe. In an extensive meta-analysis of 291,433 critically ill patients across 90 cohort studies, hypoalbuminemia was used as a predictor of outcomes using multivariable analysis. The findings showed that decreases in serum albumin levels of 10 g/L were associated with greater prolongation of hospital stay, incidence of comorbidities, and mortality rates of 71%, 89%, and 137%, respectively. A subanalysis of nine prospective controlled trials exploring treatment of hypoalbuminemia in critically ill patients showed a reduction in complications in patients with serum albumin concentrations > 30 g/L, following albumin supplementation [58]. 

Likewise, a prospective observational study involving 5894 critically ill adults showed that hypoalbuminemia at admission determined 30-day all-cause mortality independently of its prognostic mechanism [59].

In the ALBIOS study, patients with severe sepsis showed much higher 90-day mortality when serum albumin levels were less than 30 g/L at enrollment [60].

Therefore, based on current data, it is reasonable to discontinue HSA infusion if serum albumin ≥ 30 g/L and the patient is hemodynamically stable. Furthermore, the use of HSA may be considered if hemodynamic instability persists despite 30 mL/kg of crystalloid and if vascular congestion is absent.

#### 2.1.4. Optimizing Pharmacokinetics of High-Protein Binding Antibiotics

Most antibiotics given to sepsis patients lead to considerable binding of plasma protein, particularly albumin, which comprises approximately 60% of total plasma protein. Ceftriaxone sodium, ertapenem, and daptomycin are classified as highly protein-bound, with protein-binding rates of 90%, 90%, and 92%, respectively. Hypoalbuminemia, which is often observed in patients with severe septic disease, greatly impacts the pharmacokinetic characteristics of these agents; mainly the apparent volume of distribution (Vd) and clearance (CL) [61,62]. The changes in pharmacokinetics are multifaceted. Lower concentrations of albumin lead to reduced binding sites, yielding increased amounts of free (unbound) drug fractions that are pharmacologically active and undergo increased renal clearance [62]. At the same time, hypoalbuminemia lowers plasma oncotic pressure, facilitating fluid shifts from the intravascular to the interstitial space and resulting in expansion of the extracellular fluid compartment. This expansion is further enhanced by large amounts of intravenous fluids given for resuscitation in sepsis, and increases the volume of distribution [63]. The observed physiologic changes require timely titration of highly protein-binding antibiotics to enhance therapeutic efficiency and reduce the potential for toxicity in the septic population. For instance, ertapenem has a binding affinity with albumin of 85% to 95%. The Vd (0.21 ± 0.05 L/kg vs. 0.07 ± 0.03 L/kg) and the CL (43.23 ± 23.74 mL/min versus 20.21 ± 0.16 mL/min) level for ertapenem were significantly higher in patients with hypoalbuminemia and ventilator-associated pneumonia than in healthy individuals [63]. In patients with critical illness, teicoplanin binds the proteins at a rate of 90% to 95% while, in this group, its CL is higher than in healthy persons (18.2 mL/min vs. 13.4 mL/min) [64].

Based on current data, human albumin supplementation for sepsis patients receiving antibiotics with high protein-binding rates, such as ceftriaxone sodium, ertapenem, and daptomycin, is recommended to optimize pharmacokinetics and pharmacological profiles.

#### 2.1.5. Acute Respiratory Distress Syndrome (ARDS)

Fluid management in ARDS poses a significant therapeutic challenge, as the balance between ensuring adequate intravascular volume and minimizing extravascular lung water is crucial for optimizing oxygenation and organ perfusion. Albumin is the predominant plasma protein responsible for maintaining colloid oncotic pressure. It has been investigated as a potential adjunctive therapy to restore intravascular volume, attenuate capillary leak, and improve hemodynamic stability in ARDS patients [65].

In this context, evidence remains limited. Three RCTs and one meta-analysis showed no significant reduction in 28-day mortality with albumin compared with crystalloids (RR 0.77, 95% CI 0.57–1.02) [65,66,67,68]. However, albumin administration improved oxygenation in hypoalbuminemic ARDS at 24 and 48 h, and at 7 days, with weighted mean increases ranging from 20 to 62 mmHg. Improvements were not sustained beyond 72 h, where differences versus crystalloids were no longer significant [65,66].

Given the limited number of relevant studies and the relatively small sample sizes, further clinical trials are necessary to confirm these findings.

Based on the current evidence, HSA administration may be considered for patients with ARDS and hypoalbuminemia to enhance oxygenation.

#### 2.1.6. Perioperative Cardiac Surgery

Cardiac surgery is linked to hemodilution, systemic inflammation, and heightened vascular permeability, often resulting in hypoalbuminemia, decreased oncotic pressure, and tissue edema. Consequently, albumin has been utilized to restore intravascular volume, enhance hemodynamic stability, and possibly alleviate inflammatory responses [69].

In a randomized, double-blind, single-center trial involving 240 patients undergoing elective cardiac surgery, participants received either 5% human serum albumin (HSA), hydroxyethyl starch (HES), or Ringer’s lactate at a maximum dose of 50 mL·kg^−1^·day^−1^ during the perioperative phase. The findings indicated that the total fluid volume administered was lower in the HSA group compared to those receiving HES or Ringer’s lactate. However, the prevalence of patients needing blood products was significantly greater in both the HSA (62%) and HES (64%) groups when compared to the Ringer’s lactate group (35%), with a *p*-value of 0.0003 [70].

A meta-analysis encompassing 970 patients from 18 randomized controlled trials (RCTs) demonstrated that HES usage correlates with a higher incidence of blood transfusions relative to HSA. Specifically, the HES group experienced a 33.3% increase in postoperative blood loss along with increases of 28.4% in red blood cell transfusions, 30.6% in fresh frozen plasma (FFP) transfusions, and 29.8% in platelet transfusions [71].

Conversely, a substantial observational study involving 6188 adults who underwent cardiopulmonary bypass (CPB) for heart valve or coronary artery disease employed propensity score matching to assess outcomes between two groups: one receiving 5% HSA combined with crystalloid solutions and another receiving only crystalloid solutions. The results indicated that using 5% HSA alongside crystalloid solutions led to reduced rates of both in-hospital mortality and all-cause readmissions within 30 days, compared to those receiving crystalloid solutions alone [72].

Hypoalbuminemia is recognized as a predictor of negative outcomes following cardiac surgery, as it contributes to interstitial edema and renal impairment [73]. In an RCT involving 220 off-pump coronary artery bypass grafting procedures for patients with preoperative albumin levels below <40 g/L, administration of 20% albumin enhanced intraoperative urine output and diminished the overall risk of acute kidney injury (AKI) by 47%, although severe AKI rates remained unchanged [73].

Nonetheless, more recent evidence calls for caution regarding albumin use. A large-scale RCT conducted in 2025 involving 611 high-risk cardiac surgery patients revealed that postoperative administration of 20% albumin increased AKI risk when patients were given 300 mL within 6 h post-surgery over a period exceeding 15 h [74].

This finding stands in contrast to earlier studies suggesting potential renal protection for selected hypoalbuminemic patients. Overall, while albumin may help to reduce perioperative fluid requirements and could be advantageous for individuals experiencing significant hypoalbuminemia, routine postoperative use—especially concerning solutions containing 20% albumin—cannot be advised due to emerging data indicating an elevated risk for AKI.

The apparent discrepancy between earlier studies suggesting renal protection with HSA and the increased risk of acute kidney injury reported in the recent ALBICS-AKI trial may be explained by differences in timing, dose, and administration strategy. Earlier studies primarily administered HSA intraoperatively or used individualized dosing to correct hypoalbuminemia, whereas the 2025 trial evaluated a fixed postoperative bolus of hyperoncotic HSA (300 mL of 20% HSA) delivered over a short time period. In the immediate postoperative setting, such an approach may exacerbate renal vulnerability through alterations in renal perfusion pressure, increased intraglomerular oncotic pressure, or subclinical venous congestion. These findings are consistent with prior concerns regarding hyperoncotic albumin solutions in critically ill patients. In a study by Schortgen et al., administration of hyperoncotic albumin in patients with septic shock was associated with an increased risk of renal dysfunction and need for renal replacement therapy [75].

Taken together, these observations suggest that the renal effects of HSA are highly context-dependent and emphasize the importance of careful patient selection, appropriate concentration choice, and individualized dosing strategies. Accordingly, based on current evidence, albumin use should be reserved for specific clinical scenarios, particularly in patients with documented hypoalbuminemia.

#### 2.1.7. Perioperative Major Abdominal Surgery

Major abdominal surgery leads to significant physiological changes, including considerable fluid redistribution, activation of systemic inflammation, and heightened capillary permeability. These effects can result in intravascular hypovolemia and hypoalbuminemia, which negatively influence tissue perfusion, wound healing, and postoperative complications. To counteract these alterations, the exogenous administration of albumin—the primary plasma protein that helps to maintain oncotic pressure—has been utilized in the perioperative context to restore intravascular volume, reduce interstitial edema, and potentially modulate inflammatory responses.

In a randomized controlled trial (RCT) involving 100 ICU patients who had undergone abdominal surgery, participants were split into two groups: one receiving albumin infusion aimed at keeping serum albumin levels above 30 g/L, and a control group. The findings indicated that those who received albumin showed improved organ function when compared to the control group suffering from hypoalbuminemia [76].

Furthermore, a prospective observational study assessed patients with cirrhosis awaiting liver transplantation [77]. Among these individuals, 82 developed acute kidney injury (AKI) with hepatorenal syndrome (HRS) prior to transplantation and were treated with a combination of terlipressin and albumin during the study. The patients were categorized based on their treatment response—defined by a decrease of 26.5 mmol/L in serum creatinine from baseline—with 43 classified as responders and 39 as non-responders. After treatment, both groups underwent liver transplantation; each included 30 patients. Additionally, a control group of 259 patients without AKI-HRS who also had liver transplants during this timeframe was analyzed for comparison regarding transplantation outcomes. The study revealed that renal replacement therapy (RRT) was more frequently required in the non-response group than in the response group. Moreover, at one year post-transplantation, chronic kidney disease incidence was significantly higher among non-responders. Multivariate analysis indicated that not responding to terlipressin plus albumin therapy was an independent predictor of chronic renal insufficiency one year after transplant (hazard ratio [HR]: 2.76), while responders did not exhibit such an increased risk (HR: 1.53). Consequently, a favorable response to terlipressin plus albumin in AKI-HRS patients appears to reduce the necessity of RRT following liver transplantation and diminishes the likelihood of chronic renal failure within one year postoperatively [77]. 

In another RCT involving 67 patients with end-stage liver disease undergoing in situ liver transplantation, participants were divided into two groups: one received restricted fluid resuscitation along with albumin, frozen plasma, and concentrated red blood cells; the other received unrestricted fluid resuscitation at a rate of 10 mL·kg^−1^·h^−1^ saline. The results demonstrated that those treated with albumin during liver transplantation’s perioperative phase had more stable hemodynamics and experienced fewer instances of postoperative pulmonary insufficiency compared to the unrestricted fluid resuscitation group [78].

A retrospective analysis involving 998 living donor liver transplant recipients classified them based on their lowest postoperative day two albumin levels into two categories: <30 g/L (522 patients) and ≥30 g/L (476 patients). The findings indicated that individuals in the <30 g/L category experienced significantly extended ICU stays relative to those in the ≥30 g/L group [79].

A systematic review encompassing 79 RCTs with a total of 4755 participants concluded that sustaining serum protein levels above 30 g/L during the perioperative period aids in reducing additional fluid requirements, minimizing intraoperative intestinal edema, lowering clinical complications’ incidence, and safeguarding organ function [80].

Thus, the available evidence suggests that administering HSA is recommended for critically ill patients undergoing abdominal surgery when their perioperative serum albumin falls below 30 g/L, in order to maintain levels above this threshold throughout the surgical process.

#### 2.1.8. Acute Brain Injury

While crystalloids remain the preferred fluids in acute brain injury, data on albumin use in this population are inconsistent. Most large fluid-resuscitation trials include very few patients with acute brain injury, limiting generalizability. In the SAFE trial subgroup, 4% albumin was associated with higher morbidity and mortality compared with saline after traumatic brain injury (TBI) [6]. In contrast, the ALIAS pilot study suggested potential benefit of high-dose 25% albumin after ischemic stroke [81], though the larger ALIAS trial later showed no improvement in 90-day outcomes and increased rates of cerebral hemorrhage and pulmonary edema [82]. Observational studies in subarachnoid hemorrhage (SAH) report mixed results. A small single-center study [83] and a large propensity-matched cohort (*n* = 5400) [84] found improved neurological outcomes with high-dose albumin, whereas another matched analysis showed no reduction in delayed cerebral ischemia and worse NIHSS scores at 6 weeks [85]. Evidence in patients with intracerebral hemorrhage (ICH) is likewise limited to small studies suggesting potential benefits in reducing midline shift and improving EEG patterns, as well as experimental rodent data showing blood–brain barrier protection [86,87,88]. However, safety concerns persist. A reanalysis of SAFE patients with intracranial pressure (ICP) monitoring showed higher mean ICP and mortality in the albumin group during the first week after randomization, suggesting that the increased ICP may contribute to the higher mortality related to albumin [89].

HSA may exert both beneficial and detrimental effects in patients with acute brain injury, particularly in the presence of elevated intracranial pressure. From a physiological perspective, albumin can support intravascular volume, improve cerebral perfusion pressure, and stabilize the endothelial barrier, potentially limiting vasogenic edema in selected conditions such as ischemic stroke or intracerebral hemorrhage [82,83,84,85]. In addition, its antioxidant and anti-inflammatory properties may contribute to neurovascular protection under controlled circumstances [2,7].

However, in traumatic brain injury, disruption of the blood–brain barrier allows albumin to extravasate into the interstitial space, where its oncotic properties may promote cerebral edema and increase intracranial pressure [6,87,88,89]. This mechanism provides a plausible explanation for the higher ICP and increased mortality observed in the SAFE traumatic brain injury subgroup [6]. These findings underscore that the effects of albumin in acute brain injury are highly context-dependent and strongly influenced by blood–brain barrier integrity, timing of administration, and baseline intracranial dynamics.

Therefore, based on the available data, the use of albumin in TBI is not recommended. Overall, albumin may benefit selected cases of cerebral hemorrhage or ischemic stroke, but data remain heterogeneous. Large, targeted RCTs are required to clarify its role. At present, albumin is not recommended as first-line fluid therapy in acute brain injury.

#### 2.1.9. Trauma Patients

A study using data from the 2006 SAFE trial found that critically ill ICU patients, including those with trauma, had similar 28-day mortality rates after resuscitation with either HSA solution or normal saline, regardless of whether their baseline serum albumin levels were above or below 25 g/L [90].

In a systematic review of 161 trauma and surgical patients from five RCTs, the findings indicated that although HSA did not significantly reduce mortality, it was more effective than crystalloid fluids at maintaining colloid osmotic pressure. This, in turn, helped to reduce intestinal edema and improved cardiac output in trauma and surgical patients, contributing to better circulatory stability [91].

Based on the available evidence, HSA may be considered for use in trauma patients who have severe hypoalbuminemia and/or unstable hemodynamics.

#### 2.1.10. Patients with Extracorporeal Membrane Oxygenation (ECMO)

Hypoxia and hypoperfusion can often lead to capillary leakage and a loss and redistribution of intravascular volume. Reoxygenation or reperfusion during the early stages of ECMO can exacerbate this situation, resulting in insufficient circulating blood volume in the right heart and blood vessels. Fluid resuscitation is necessary during the early phase of ECMO [92]. However, there remains controversy regarding the optimal fluid resuscitation strategy during ECMO support.

In one retrospective study involving 283 patients on veno-arterial ECMO, a positive fluid balance at 12 h after cannulation was associated with better in-hospital survival in patients receiving a combination of human serum albumin (HSA) (10 g of albumin per liter) and crystalloids (1:2 ratio), compared to those who received crystalloids alone. Multivariate logistic regression analysis indicated that combining HSA and crystalloids improved in-hospital survival, with an OR of 3.10 (95% CI: 1.15–6.38) after PSM. Subgroup analysis suggested that the benefit was especially significant in elderly patients with hyperlactatemia, a low Sequential Organ Failure Assessment (SOFA) score, and a lower survival after veno-arterial ECMO (SAVE) score [93].

Based on the available data, the combination of HSA and crystalloids may be considered as an effective fluid resuscitation strategy for patients undergoing ECMO.

Albumin dosing strategies varied substantially across clinical trials, depending on indication, study design, and therapeutic goals. Table 2 summarizes the dosing regimens reported in the clinical trials included in this review.

#### 2.1.11. Effectiveness, Safety, Adverse Effects, and Pharmacological Considerations

The clinical effectiveness of HSA is highly indication-dependent. In end-stage liver disease, albumin improves renal and circulatory outcomes in selected scenarios such as SBP, HRS, and prevention of PICD [37,41,42,43]. In contrast, in critical illness and perioperative settings, large randomized trials have shown neutral overall mortality compared to crystalloids, with potential benefits limited to specific subgroups such as patients with septic shock, and heterogeneous results in surgical populations [6,46,47,70,74]. In acute brain injury, HSA use is associated with increased ICP and mortality in traumatic brain injury, whereas data in ischemic stroke, subarachnoid hemorrhage, and intracerebral hemorrhage remain inconsistent [6,82,83,84,85,86,90].

From a safety perspective, HSA is generally well tolerated; however, clinically relevant adverse effects include fluid overload and pulmonary edema, particularly with rapid infusion, higher concentrations (20–25%), or in patients with limited cardiac or renal reserve [2,7]. Emerging evidence also suggests that the administration strategy and timing influence renal safety, as postoperative hyperoncotic albumin has been associated with an increased risk of acute kidney injury in cardiac surgery [74]. In acute brain injury, disruption of the BBB may allow for albumin extravasation, potentially worsening cerebral edema and ICP [6,87,88,89,90].

Potential drug interactions with HSA are primarily pharmacokinetic. Albumin is the main plasma binding protein for many drugs; therefore, hypoalbuminemia and albumin supplementation may alter the free fraction of highly protein-bound medications, with potential implications for efficacy and toxicity, particularly in critically ill patients [2,7,62,63,64,65]. These effects are especially relevant for drugs with narrow therapeutic windows, underscoring the importance of clinical monitoring and, when available, therapeutic drug monitoring based on free drug concentrations.

## 3. Conclusions

In conclusion, human serum albumin has been used in clinical practice since the 1940s. Its ability to maintain colloid osmotic pressure, provide antioxidant effects, and regulate nitric oxide makes it an important therapeutic option in critical care. Nevertheless, its role in the ICU remains debated, as its benefits vary across different patient groups. Future research should aim to identify which critically ill patients are most likely to benefit from albumin therapy and address several clinically relevant questions that currently rest on limited evidence. Alongside large, well-designed randomized controlled trials, further mechanistic studies are required to clarify the molecular and physiological pathways through which albumin exerts its effects. Additional priorities include understanding the causes of hypoproteinemia in critical illness, defining optimal albumin dosing and treatment targets, and determining whether different albumin concentrations result in distinct clinical outcomes.

Furthermore, current evidence suggests that iso-oncotic albumin solutions (4–5%) should primarily be used for intravascular volume replacement, whereas hyperoncotic formulations should be reserved for selected indications and administered with caution [94].

The findings of this review should be interpreted in light of several limitations. As a narrative review, no formal systematic screening process was applied, and the available evidence is characterized by substantial heterogeneity in study design, patient populations, albumin concentration, dosing strategies, and outcome measures. These factors limit direct comparisons across clinical indications. Future research should therefore focus on indication-specific, adequately powered randomized controlled trials to better define patient selection and the timing, dose, and concentration of albumin therapy, as well as to further clarify its safety profile in vulnerable populations.

## Figures and Tables

**Table 1:** Summary of meta-analyses and randomized trial evaluating albumin-based therapies in HRS.

Study	Design	Comparison	Outcome	Effect Measure	Estimate	95% CI	Mortality Effect
Facciorusso 2017 [26]	Network meta-analysis (13 RCTs)	Albumin + Terlipressin vs Albumin alone	3-month mortality	OR	0.65	0.41–1.05	Not significant
Nanda 2018 [27]	Meta-analysis (13 RCTs)	Terlipressin + Albumin vs Albumin alone [24,28,29,30]	HRS reversal	OR	4.72	1.72–12.93	No mortality benefit
		Terlipressin + Albumin vs Midodrine + Octreotide + Albumin [31]	HRS reversal	OR	5.94	1.69–20.85	
Best 2019 [32]	Network meta-analysis (25 RCTs)	Terlipressin + Albumin vs Midodrine + Octreotide + Albumin	HRS recovery	HR	0.04	0.00–0.25	No mortality benefit
China 2021 [33]	RCT	Additional Albumin vs Standard care	AKI incidence	Adjusted OR	0.68	0.44–1.10	Not significant

RCT: Randomized Clinical Trial; OR: Odds Ratio; HR: Hazard Ratio; HRS; Hepatorenal syndrome; AKI; Acute Kidney Injury.

**Table 2:** HSA dosing regimens in Clinical Trials.

Clinical Condition	Study-Clinical Trial	HSA Concentration	Dosing Regimen Reported in the Trial
SBP	AASLD Practice Guidance—Biggins et al. [36]	20–25%	1.5 g/kg on day 1 followed by 1 g/kg on day 3, as reported in the AASLD practice guidance
HRS	Boyer et al., Gastroenterology 2016 [24]	20–25%	Albumin administered in combination with terlipressin (initial loading followed by daily dosing; typically 1 g/kg day 1, then 20–40 g/day)
	Wong et al., NEJM 2021 [25]	20–25%	1 g/kg on day 1 (maximum 100 g), followed by 20–40 g/day during vasoconstrictor therapy
Large-volume paracentesis (>5 L)	Bernardi et al., Hepatology 2012 [40]	20–25%	6–8 g of albumin per liter of ascites removed, typically starting from the fifth liter
	Simonetti et al., Cochrane Review [42]	20–25%	Same volume-adjusted dosing per liter removed
Modest-volume paracentesis (≤5 L)	Bernardi et al., Hepatology 2012 [40], Simonetti et al., Cochrane Review [42]	20–25%	Fixed doses of 20–40 g or volume-adjusted dosing, depending on study protocol
Septic shock	SAFE trial—Finfer et al. [6]	4%	Albumin used for fluid resuscitation as clinically indicated (no fixed dosing regimen)
	ALBIOS trial—Caironi et al. [47]	20%	Daily albumin infusion to maintain serum albumin ≥ 30 g/L until ICU discharge or day 28
ARDS	Martin et al., Crit Care Med 2002 [66]	25%	25 g every 8 h for 72 h, combined with diuretics, in hypoalbuminemic patients
	Martin et al., Crit Care Med 2005 [65]	25%	Same dosing strategy (25 g every 8 h for 72 h)
Periop. cardiac surgery	Skhirtladze et al., Br J Anaesth 2014 [70]	5%	Albumin administered intra- and postoperatively up to a maximum of 50 mL·kg^−1^·day^−1^
	Shehabi et al., ALBICS-AKI trial [74]	20%	Postoperative infusion of 300 mL of 20% albumin within the first 6 h after surgery
Periop. major abd. surgery	Dubois et al., Crit Care Med 2006 [76]	20–25%	Albumin administered to correct hypoalbuminemia and maintain serum albumin ≥ 30 g/L (no fixed dose)
	Sahmeddini et al., Int J Organ Transplant Med 2014 [78]	20–25%	Albumin used as part of a restricted fluid strategy during liver transplantation (dose not protocolized)
Acute brain injury	SAFE trial—TBI subgroup [6]	4%	Albumin used as resuscitation fluid without predefined dosing
	ALIAS trials—Palesch et al. [80], Martin et al. [82]	25%	High-dose albumin up to 2 g/kg administered within 48 h after ischemic stroke
Trauma patients	SAFE trial—trauma subgroup [90]	4%	Albumin administered for volume resuscitation according to clinical need (no fixed dosing regimen)
Patients on ECMO	Wengenmayer et al., Intensive Care Med 2018 [93]	5%	Albumin added to crystalloids at approximately 10 g per liter of infused fluid during early VA-ECMO resuscitation

SBP: Spontaneous bacterial peritonitis; HRS: Hepatorenal syndrome; ARDS: Acute respiratory distress syndrome.

## Data Availability

No new data were created or analyzed in this study.

## Abbreviations

AKI	acute kidney injury;
ARDS	acute respiratory distress syndrome;
BBB	blood–brain barrier;
BUN	blood urea nitrogen;
CKD	chronic kidney disease;
CPB	cardiopulmonary bypass;
CPP	cerebral perfusion pressure;
ECMO	extracorporeal membrane oxygenation;
EEG	electroencephalography;
FFP	fresh frozen plasma;
HES	hydroxyethyl starch;
HRS	hepatorenal syndrome;
HRS-AKI	hepatorenal syndrome–acute kidney injury;
HSA	human serum albumin;
ICH	intracerebral hemorrhage;
ICP	intracranial pressure;
ICU	intensive care unit;
LVP	large-volume paracentesis;
NIHSS	National Institutes of Health Stroke Scale;
PICD	paracentesis-induced circulatory dysfunction;
PSM	propensity score matching;
RCT	randomized controlled trial;
RRT	renal replacement therapy;
SAH	subarachnoid hemorrhage;
SAVE	Survival After Veno-arterial ECMO score;
SBP	spontaneous bacterial peritonitis;
SIRS	systemic inflammatory response syndrome;
SOFA	Sequential Organ Failure Assessment;
SV	stroke volume;
TBI	traumatic brain injury;
VA-ECMO	veno-arterial extracorporeal membrane oxygenation;
Vd	volume of distribution.
